# The Treatment of Cancer

**Published:** 1904-12-10

**Authors:** 


### THE TREATMENT OF CANCER.

In the Bradshaw Lecture, which he delivered last week, Mr. Mayo Robson was happy in his choice of a subject, and happier still in his method of dealing with it. The chief feature of the address was his protest "both against the fatalistic tendency to delay and the senseless running after false gods in the treatment of this fell disease." "Too late" has yet to be said in one-half or three-fourths of the cancer cases when seen by the operating surgeon. Thus surgery is deprived of the opportunity of achieving that success of which it is really capable. Early and complete removal with the knife can offer a fair prospect of cure, and further than this a great deal more could be done by a more frequent resort to "preventive operations." In certain situations, such as the tongue, lips, larynx, uterus, and the skin, pre-cancerous conditions can be recognised; and it is probable that every cancer, whether external or internal, follows on a pre-cancerous condition—such, for example, as cancer of the gall-bladder following on ulceration produced by gall-stones, cancer of the stomach on chronic gastric ulcer, epithelioma of the penis on irritation under a phimosis, cancer of the bladder on papilloma or on ulcers due to calculi, and cancer of the rectum and colon on stercoral or other ulcers. The liability of benign tumours, especially on epithelial surfaces, to undergo malignant changes is well recognised, hence the removal of such is generally advisable. A general acceptance of the view that cancer has usually a pre-cancerous stage and that this stage is the one in which an operation ought to be performed would be the means of saving many lives. Again, preventive treatment includes the removal of known factors in the causation of cancer as well as the abolition of discoverable pre-cancerous conditions. Thus excessive smoking, especially when a clay-pipe is used, sharp edges of teeth, and ill-fitting and irritating artificial tooth-plates are all recognised as taking part in the production of oral cancer, and they are all factors which can be avoided.

We can join with Mr. Robson in the hope that the necessity for anticipating malignant growths will be realised one day by the public, and that they will cease to trifle with their ailments until the favourable moment has gone by, and they can only receive the verdict, too often pronounced, Too late.

